# Abacavir Exposure in Children Cotreated for Tuberculosis with Rifampin and Superboosted Lopinavir-Ritonavir

**DOI:** 10.1128/AAC.01923-19

**Published:** 2020-04-21

**Authors:** Helena Rabie, Tjokosela Tikiso, Janice Lee, Lee Fairlie, Renate Strehlau, Raziya Bobat, Afaaf Liberty, Helen McIlleron, Isabelle Andrieux-Meyer, Mark Cotton, Marc Lallemant, Paolo Denti

**Affiliations:** a Family Centre for Research with Ubuntu, Stellenbosch University, Cape Town, South Africa; b Department of Paediatrics and Child Health, Stellenbosch University and Tygerberg Hospital, Cape Town, South Africa; c Division of Clinical Pharmacology, Department of Medicine, University of Cape Town, Cape Town, South Africa; d Drugs for Neglected Diseases initiative, Geneva, Switzerland; e Wits Reproductive Health and HIV Institute, Faculty of Health Sciences, Department of Paediatrics and Child Health, University of the Witwatersrand, Johannesburg, South Africa; f Empilweni Services and Research Unit, Rahima Moosa Mother and Child Hospital, Department of Paediatrics and Child Health, University of the Witwatersrand, Johannesburg, South Africa; g Enhancing Care Foundation—Durban International Clinical Research, Wentworth Hospital, Durban, South Africa; h Perinatal HIV Research Unit, University of the Witwatersrand, Johannesburg, South Africa

**Keywords:** abacavir, rifampin, children, lopinavir, NONMEM, population pharmacokinetics

## Abstract

In children requiring lopinavir coformulated with ritonavir in a 4:1 ratio (lopinavir-ritonavir-4:1) and rifampin, adding ritonavir to achieve a 4:4 ratio with lopinavir (LPV/r-4:4) overcomes the drug-drug interaction. Possible drug-drug interactions within this regimen may affect abacavir concentrations, but this has never been studied. Children weighing <15 kg needing rifampin and LPV/r-4:4 were enrolled in a pharmacokinetic study and underwent intensive pharmacokinetic sampling on 3 visits: (i) during the intensive and (ii) continuation phases of antituberculosis treatment with LPV/r-4:4 and (iii) 1 month after antituberculosis treatment completion on LPV/r-4:1. Pharmacometric modeling and simulation were used to compare exposures across weight bands with adult target exposures. Eighty-seven children with a median (interquartile range) age and weight of 19 (4 to 64) months and 8.7 (3.9 to 14.9) kg, respectively, were included in the abacavir analysis. Abacavir pharmacokinetics were best described by a two-compartment model with first-order elimination and transit compartment absorption. After allometric scaling adjusted for the effect of body size, maturation could be identified: clearance was predicted to be fully mature at about 2 years of age and to reach half of this mature value at about 2 months of age. Abacavir bioavailability decreased 36% during treatment with rifampin and LPV/r-4:4 but remained within the median adult recommended exposure, except for children in the 3- to 4.9-kg weight band, in which the exposures were higher. The observed predose morning trough concentrations were higher than the evening values. Though abacavir exposure significantly decreased during concomitant administration of rifampin and LPV/r-4:4, it remained within acceptable ranges. (This study is registered in ClinicalTrials.gov under identifier NCT02348177.)

## Abstract (precis)

In children requiring lopinavir coformulated with ritonavir in a 4:1 ratio (lopinavir-ritonavir-4:1) and rifampin, adding ritonavir to achieve a 4:4 ratio with lopinavir (LPV/r-4:4) overcomes the drug-drug interaction. Possible drug-drug interactions within this regimen may affect abacavir concentrations, but this has never been studied. Children weighing <15 kg needing rifampin and LPV/r-4:4 were enrolled in a pharmacokinetic study and underwent intensive pharmacokinetic sampling on 3 visits: (i) during the intensive and (ii) continuation phases of antituberculosis treatment with LPV/r-4:4 and (iii) 1 month after antituberculosis treatment completion on LPV/r-4:1.

## TEXT

In HIV-positive children younger than 3 years of age, the World Health Organization (WHO) recommends the nucleoside reverse transcriptase inhibitor (NRTI) abacavir in the first-line antiretroviral therapy (ART) regimen with lamivudine and the protease inhibitor (PI) lopinavir coformulated with ritonavir (LPV/r) in a 4:1 ratio (lopinavir-ritonavir-4:1 or LPV/r-4:1) (1). WHO indicates abacavir for children from 3 months of age at 8 mg/kg of body weight twice daily or 16 mg/kg once daily to a maximum of 300 mg per dose twice daily or 600 mg daily (2, 3). In South Africa, abacavir is recommended from the age of 1 month and a weight above 3 kg (4). The risk for abacavir hypersensitivity is related to genetic factors and is very low in African children (5). Abacavir is extensively metabolized by the liver, with less than 2% being excreted unchanged in urine (6). The two major pathways of abacavir metabolism are the alcohol dehydrogenase (ADH) and UDP glucuronyltransferase (UGT) enzymes, producing the inactive metabolites carboxylate and glucuronide, respectively (6). Coadministration with food has no effect on abacavir exposure (7). Abacavir pharmacokinetic parameters do not appear to be affected by the use of a liquid or a tablet formulation (8).

Lopinavir coformulated with ritonavir in a 4:1 ratio (lopinavir-ritonavir-4:1) is superior to nevirapine in young infants, regardless of the nevirapine exposure, as part of the prevention of mother-to-child transmission (9, 10). The low dose of ritonavir inhibits the cytochrome P450 3A4 (CYP3A4)-mediated metabolism of lopinavir and the P-glycoprotein (P-gp) efflux pump, thereby providing effective lopinavir plasma exposure (11). Ritonavir may also induce some cytochrome P450 enzymes and various glucuronidases (UGTs) and multidrug transport proteins, such as P-glycoprotein (12–16). Adult data suggest that the coadministration of abacavir and lopinavir-ritonavir-4:1 has no effect on lopinavir pharmacokinetics (PK) but appears to decrease abacavir exposure by approximately 30% (17). Nevertheless, no dose adjustments are recommended, and the significance of this drug-drug interaction is not known.

The epidemiology of tuberculosis (TB) and HIV overlaps, particularly in sub-Saharan Africa, and TB infection remains common in HIV-positive children (18). The short-course regimens for drug-susceptible TB in children consist of daily rifampin, isoniazid, and pyrazinamide with or without ethambutol or ethionamide for 2 months, followed by isoniazid and rifampin for 4 months. Rifampin induces CYP3A4 and is a strong activator of the pregnane X receptor (PXR), which upregulates many drug-metabolizing enzymes and drug transporters, leading to major drug-drug interactions when administered with antiretrovirals metabolized by the same pathways. Thus, rifampin induces the expression of P-gp and UGT, responsible for abacavir metabolism (19, 20). Conversely, rifampin inhibits hepatic organic anion-transporting polypeptides (OATPs). Lopinavir exposure is decreased by up to 90% when administered with rifampin (21). To overcome this drug-drug interaction, adding ritonavir to achieve a 4:4 ratio with lopinavir (LPV/r-4:4; also referred to as superboosting) is a commonly used strategy to overcome the induction by rifampin, thereby providing effective plasma concentrations of lopinavir. There are no pharmacokinetic data available on the implications of LPV/r-4:4 and rifampin on abacavir exposure.

We conducted a large pharmacokinetic study to confirm the validity of the LPV/r-4:4 approach in young children with TB and HIV. Within this study, we also investigated abacavir pharmacokinetics during treatment with LPV/r-4:4 with rifampin and during standard lopinavir-ritonavir-4:1 treatment after the end of rifampin treatment.

## RESULTS

Between January 2013 and November 2015, we enrolled 96 children into the main study (22). However, not all children received abacavir and/or completed the study. Eighty-seven children were included in the abacavir analysis, with 86 PK profiles being obtained in the first PK visit, 74 in the second PK visit, and 71 in the third PK visit. A total of 1,344 measured drug concentrations were available for analysis, and 18 samples were excluded from the analysis due to an unclear dosage history or concentrations incoherent with the recoded dosing. The patient characteristics are shown in Table 1. The median (interquartile range) age and weight at enrollment were 19 (4 to 64) months and 8.7 (3.9 to 14.9) kg, respectively. Children younger than 12 months were well represented in this cohort, with 8% (6/71) of the children still being younger than 12 months at the third PK visit. The majority of patients at each PK visit weighed between 5 and 9.9 kg. At the first PK visit, 7 (8%) patients were in the 3- to 4.9-kg weight group.

**TABLE 1:** Clinical characteristics of patients at each pharmacokinetic visit

Characteristic^*a*^	Value at:		
	PK visit 1	PK visit 2	PK visit 3
No. of patients in analysis/no. of male patients (%)	86/37 (43)	74/29 (36)	71/27 (39)
No. of samples in analysis/no. of samples with values BLQ (%)	504/84 (17)	436/59 (13)	404/45 (11)
Median (IQR) age (mo)	19 (4 to 64)	23 (8 to 68)	26 (10 to 70)
No. (%) of patients <1 yr of age	25 (30)	13 (17)	6 (8)
Median (IQR) wt (kg)	8.7 (3.9 to 14.9)	9.6 (5.7 to 15.9)	10.2 (6.8 to 15.9)
No. (%) of patients in the following wt band (kg):			
3–4.9	7 (8)	Nil	Nil
5–9.9	51 (59)	40 (54)	35 (50)
10–13.9	24 (28)	26 (35)	26 (36)
14–19.9	4 (5)	8 (11)	10 (14)
Z-score^*b*^			
Wt-for-age	−2.43 (−5.19 to 1.34)	−1.93 (−4.84 to 1.55)	−1.50 (−4.86 to 1.51)
Wt-for-ht	−2.02 (−4.14 to 2.25)	−1.80 (−3.68 to 4.28)	−1.12 (−3.74 to 4.31)
Median (IQR) dose (mg/kg)			
Abacavir BID	9.52 (7.84 to 12.8)	9.72 (7.62 to 16.3)	9.60 (7.79 to 13.2)
Rifampin OD	14.6 (10.2 to 15.0)	11.9 (11.6 to 13.6)	Nil
Lopinavir BID	14.5 (11.5 to 23.1)	13.8 (11.0 to 21.1)	13.5 (11.4 to 18.5)
Ritonavir (total) BID	15.1 (11.7 to 24.2)	14.2 (11.0 to 22.1)	3.3 (2.86 to 4.62)

a IQR, interquartile range; OD, once a day; BID, twice a day.

b Z-scores were calculated according to the WHO growth chart.

**FIG 1:**
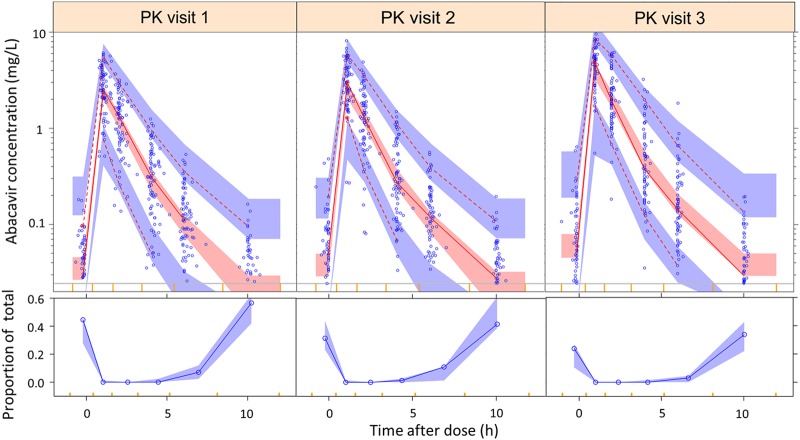
(Top) Visual predictive check of abacavir concentration versus time after dose, stratified by PK visit. PK visit 1 is the intensive phase of antituberculosis treatment with LPV/r-4:4, PK visit 2 is the continuation phase of antituberculosis treatment with LPV/r-4:4, and PK visit 3 represents 1 month after antituberculosis treatment completion on LPV/r-4:1. The solid and dashed lines represent the 50th, 5th, and 95th percentiles of the observed data, while the shaded areas represent the model-predicted 95% confidence intervals for the same percentiles. The dots are the observed concentrations. The yellow ticks on the *x* axis are bin boundaries. (Bottom) Proportion of LLOQ values versus time after dose. The solid blue line represents the observed proportion, while the blue shaded area is the 90% confidence interval for the same proportion, as predicted by the model.

Abacavir pharmacokinetics was best described by a two-compartment disposition model (difference in objective function value [ΔOFV] = −728 compared to a one-compartment model, *P < *10^−6^), with first-order elimination and transit compartments describing absorption (ΔOFV = −148 compared with simple first-order absorption, *P < *10^−6^). The model fitted the data well, as evident from the visual predictive checks (VPC) shown in Fig. 1. All parameter estimates are shown in Table 2.

**TABLE 2:** Final parameter estimates for abacavir population pharmacokinetic model

Model parameter	Typical value		Variability^*a*^	
	Value	95% CI^*b*^	% CV	95% CI
Clearance (liters/h)^*c*^	9.67	8.27 to 10.6	14.4 (BSV)	10.9 to 18.5
			18.4 (BVV)	14.4 to 21.4
Central vol of distribution (liters)^*c*^	8.76	6.99 to 9.94		
Absorption rate constant *k_a_*; (1/h)	2.22	1.82 to 2.80	55.5 (BOV)	47.0 to 72.0
Bioavailability	1 (fixed)		44.8 (BOV)	32.9 to 45.9
Peripheral vol of distribution (liters)^*c*^	3.32	2.82 to 3.98	29.0 (BSV)	19.2 to 33.6
Intercompartmental clearance (liters/h)^*c*^	1.35	1.09 to 1.67	13.5 (BSV)	12.4 to 19.9
γ^*d*^	4.35	3.23 to 5.50		
PMAGE_50_^*e*^ (time [mo] from conception)	10.7	10.5 to 11.0		
Absorption mean transit time (min)^*f*^	3.60	2.64 to 6.54	175 (BOV)	141 to 205
No. of transit compartments	1.03	0.901 to 1.20		
Proportional error (%)	23.0	21.2 to 25.0		
Additive error (μg/liter)	0.310	0.0661 to 0.400		
LPV/r-4:4 + rifampin effect on bioavailability (%)	−36.0	−38.7 to −32.9		
Delay in absorption for night dose (h)	2.62	2.23–2.90	9.90 (BVV)	9.52 to 15.4

a Between-subject variability (BSV), between-visit variability (BVV), and between-occasion variability (BOV) variability were assumed to be lognormally distributed and are reported as the coefficient of variation (CV).

b The 95% confidence interval (CI) of the parameter estimates was obtained with sampling importance resampling (SIR; *n* = 1,000) of the final model.

c All clearances and volumes of distribution were allometrically scaled, and the typical values reported here refer to a child weighing 9.4 kg, the median value in the data set.

d γ is the shape factor in the sigmoidal maturation function.

e PMAGE_50_ is the postmenstrual age at which 50% maturation is reached.

f The absorption mean transit time is the average time that the drug spends traveling from the first transit compartment to the absorption compartment.

**FIG 2:**
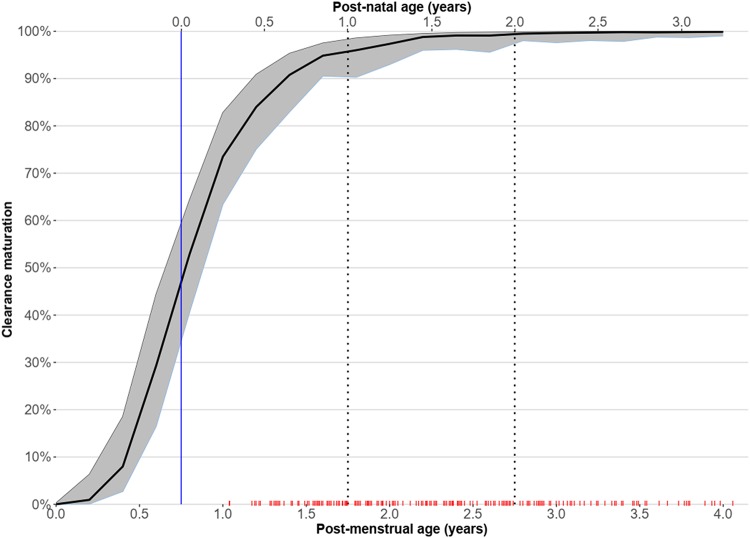
Maturation function of abacavir clearance versus postmenstrual age (bottom *x* axis) or postnatal age (top *x* axis; assuming an average gestation of 9 months), after adjusting for weight. The solid vertical blue line represents birth, while the dashed vertical lines represent 1 year and 2 years of postnatal age. The red ticks on the lower *x* axis represent the postmenstrual age values available in our data.

The inclusion of allometric scaling with total body weight (TBW) improved the model fit (ΔOFV = −38), and the use of fat-free mass (FFM) instead did not provide any further meaningful improvements. After adjusting for body size, we identified a maturation effect on abacavir clearance (ΔOFV = −14, *P < *10^−3^), which was predicted to reach to full maturation by about 2 years of age and half of its mature value at about 2 months of age, as shown in Fig. 2.

The typical abacavir clearance for a 9.4-kg child cotreated with LPV/r at the standard 4:1 dose was estimated to be 9.67 liters/h. Importantly, a 36% decrease in bioavailability (and, thus, overall exposure) of abacavir was noted during coadministration of rifampin and LPV/r-4:4 (ΔOFV = −44, *P < *10^−6^). Since the coadministration of rifampin coincided with ritonavir superboosting in our data set, we tried to use ritonavir exposure (we calculated individual area under the concentration-time curve [AUC] values with noncompartmental analysis techniques) as an alternative predictor to explain the decrease in abacavir bioavailability (results not shown). Ritonavir exposure was very variable, and although the concentration during the superboosting dose was indeed higher, there was a wide overlap with the exposure while on standard LPV/r-4:1. However, not only did the use of ritonavir exposure not improve the model fit compared to that achieved by use of the categorical covariate rifampin coadministration, but also the model unintuitively identified a weak positive correlation between the exposure of abacavir and ritonavir, which is the contrary of what would be expected if a larger increasing ritonavir AUC were responsible for the lower bioavailability of abacavir.

Additionally, we found that the observed morning predose concentrations (*C*_0_) were larger than expected if a perfect 12-h steady-state scenario is assumed. In fact, the observed morning predose concentrations were larger than the observed concentration at 10 h (*C*_10_), as shown in Table 3. To account for this, an absorption delay parameter was included in the model for the unobserved doses given to the children on the evening before the PK visit. This improved the model fit significantly (ΔOFV = −127, *P < *10^−6^) and adjusted for this difference.

**TABLE 3:** Abacavir exposures and predose concentrations in each PK visit

Model parameter	Value at^*a*^:		
	PK visit 1	PK visit 2	PK visit 3
Model-predicted AUC_0–12_ (mg·h/liter)	6.36 (1.28 to 14.2)	7.01 (1.46 to 16.5)	10.2 (2.08 to 25.6)
Model-predicted *C*_max_^*b*^ (mg/liter)	3.32 (0.741 to 6.29)	3.34 (1.44 to 7.05)	4.93 (1.75 to 11.1)
Observed *C*_max_ (mg/liter)	2.52 (0.462 to 6.07)	2.95 (0.274 to 8.12)	4.87 (0.551 to 11.5)
Observed *C*_0_ (mg/liter)	0.0282 (BLQ to 0.181 [44.6]^*c*^)	0.0433 (BLQ to 0.480 [35.6])	0.0444 (BLQ to 0.641 [23.8])
Observed *C*_10_ (mg/liter)	BLQ (BLQ to 0.162 [56.1])	0.026 (BLQ to 0.195 [41.6])	0.0308 (BLQ to 0.195 [40.3])

a The data are reported as the median (IQR).

b *C*_max_, maximum concentration in plasma.

c The percentage of the observed values below the limit of quantification is provided in square brackets.

### Dosing simulations.

**FIG 3:**
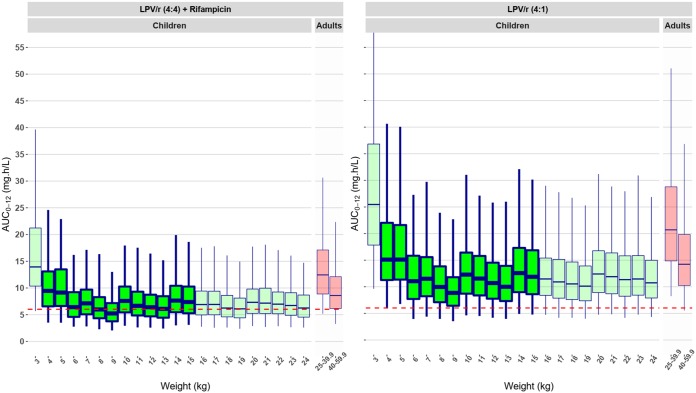
Simulated steady-state abacavir AUC_0–12_ versus body weight. (Left) Exposures during cotreatment with superboosted LPV/r-4:4 and rifampin; (right) exposures during cotreatment with unboosted LPV/r-4:1. The box indicates the interquartile range, while the whiskers denote the 2.5th and the 97.5th percentiles. The green box plots show the exposures of children weighing from 3 to 24.9 kg receiving the current pediatric dosing, as shown in Table 4, while the red box plots show the predicted exposure in adults weighing 25 to 39.9 kg and 40 to 59.9 kg, with children receiving a dose of 300 mg twice daily and adults receiving a dose of 600 mg once daily. The adult AUC_0–24_ was divided by 2 to obtain a value comparable to the AUC_0–12_ for comparison to the children’s exposures. The red horizontal dashed line represents the recommended median adult exposure (6.02 mg·h/liter). The weights of the children in this study population were mostly in the range of 4 to 16 kg; all the results outside this weight range (boxes with faint color) were extrapolated using maturation and allometric scaling. To improve the readability of the chart, the *y* axis was cut; the 97.5th percentile predicted AUC for children in the 3-kg weight band reached 67 mg·h/liter.

The simulated pediatric exposures, which were compared to the target recommended 12-h adult median exposure (AUC from 0 to 12 h [AUC_0–12_] of 6.02 mg·h/liter), are shown in Fig. 3. When cotreated with LPV/r-4:4, the simulated exposures in most children’s weight groups were in line with the adult target, with the exception of those in children in the 3- to 4.9-kg weight group, in which the exposures were significantly higher than those in children in the other weight groups. When treated with standard doses of LPV/r-4:1, all pediatric weight groups had higher exposures than the recommended adult median exposure, with the same trend of an even higher exposure in the 3- to 4.9-kg weight group being seen.

The model was also used to simulate the predicted concentrations in adolescents and adults by using weights of 25 to 59.9 kg and the currently recommended 600-mg-once-daily adult dose. The AUC from 0 to 24 h (AUC_0–24_) was divided by 2 to obtain a value comparable to the AUC_0–12_ used in children. The model predicted that adults with weights of 40 to 59.9 kg achieve exposure in line with that of most of the children weighing 6 to 24 kg, but due to the fact that all adults receive the same dose, the subjects with weights of 25 to 39.9 kg had substantially higher exposures.

In summary, all children achieved values in line with or larger than the recommended target. When comparing the children and the extrapolated adult exposures from our model, we can then conclude that most children weighing 5 to 24.9 kg achieve an exposure in line with that for adults weighing 40 to 59.9 kg, while both young children weighing 3 to 4.9 kg and young adults weighing 25 to 39.9 kg achieve higher concentrations.

## DISCUSSION

We developed a population PK model of abacavir in children characterizing the effects of weight and age. Our model identified a significant 36% reduction in abacavir exposure when the children were cotreated with rifampin and LPV/r-4:4. In the parent study, we showed that superboosting of LPV/r from LPV/r-4:1 to LPV/r-4:4 achieves similar lopinavir concentrations (22), so it is unclear whether the decreased exposure was due to the extra ritonavir added to boost LPV/r-4:4 or the rifampin cotreatment. Both ritonavir and rifampin induce UGT and P-glycoprotein. However, when ritonavir exposure was tested as an alternative predictor in the model to explain the lower bioavailability of abacavir, it could not explain the observed effect. This suggests that the effect is probably related to rifampin, but further investigation is needed to confirm this.

**FIG 4:**
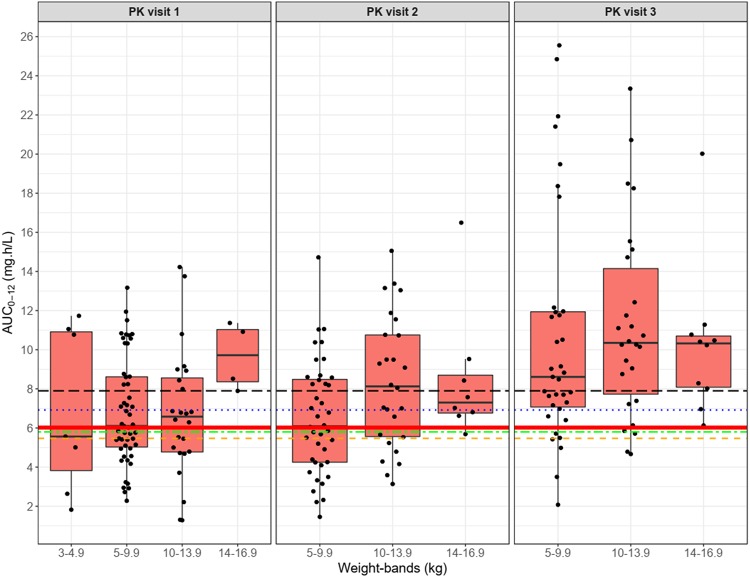
Summary of model-predicted abacavir AUC_0–12_ versus weight bands in each pharmacokinetic visit. The box indicates the interquartile range, while the whiskers denote the 2.5th and the 97.5th percentiles. Each dot represents an individual AUC. PK visit 1 is the intensive phase of antituberculosis treatment with LPV/r-4:4, PK visit 2 is the continuation phase of antituberculosis treatment with LPV/r-4:4, and PK visit 3 represents 1 month after the completion of antituberculosis treatment with LPV/r-4:1. The red horizontal solid line represents the reference median AUC, while the broken lines represent adult AUC values from the literature: Yuen et al. (7) (dashed orange line), Moyle et al. (17) (long-dash black line), McDowell et al. (6) (dot-dash green line), and Weller et al. (43) (dotted blue line).

The coadministration of abacavir and LPV/r-4:1 does not affect lopinavir plasma concentrations but is thought to reduce abacavir levels by approximately 30%, presumably through increased glucuronidation, as with other protease inhibitors (23). In adults, the clinical significance of this drug-drug interaction is not known. However, in children there was some early indication that stavudine- and LPV/r-4:1-based regimens may outperform abacavir- and LPV/r-4:1-based regimens and that this could be due to this drug-drug interaction, but subsequent data did not confirm this finding (24). In this cohort of children, cotreatment with abacavir and LPV/r-4:1 did not lead to unexpected abacavir levels, as shown in Fig. 4, but we did not compare the level achieved to that found in children on nonnucleoside reverse transcriptase inhibitors or integrase inhibitors.

The simulations show that although children cotreated with LPV/r-4:4 and rifampin had reduced abacavir exposure, AUC_0–12_ was comparable to the adult target, and their exposures were comparable to those seen in other studies conducted in children (25–28). Young infants are predicted to achieve even higher exposures, due to immature metabolic pathways. Although abacavir is now the first-line treatment for most young children in low-resource settings, it is currently not licensed for use in children below the age of 3 months. In South Africa, it is used in children over 1 month of age, despite a lack of data. Our abacavir PK model showed that a dose of 8 mg/kg twice daily is adequate for children cotreated with LPV/r-4:4 and rifampin starting at a weight of 3 kg to maintain a clinically relevant exposure and is higher in children cotreated with abacavir and LPV/r-4:1. The results for children in the 3- to 4.9-kg weight band should be interpreted with caution. Only 7 study subjects were in this weight band, and their AUCs were very variable, with some very low values being observed. Besides the effect of maturation of clearance, other factors may introduce variability in drug concentrations in these small children, including difficulties with drug administration. Better characterization of pharmacokinetics in children younger than 3 months of age is needed to determine a dose with more certainty and, consequently, allow incorporation into neonatal and infant treatment protocols. We had very few young children, so the characterization of maturation has limited precision (Fig. 2). Additionally, information on weight band approaches to dosing in the smallest and youngest infants is also lacking.

The observed abacavir *C*_0_ were higher than the observed *C*_10_. A diurnal effect with higher modeled morning trough lopinavir concentrations was also shown in this cohort (22). Similarly, in a study by Van Heeswijk et al., LPV/r given in the evening or in the morning had the same AUC (thus, not much of a difference in clearance), but the profile was delayed, with the observed concentration at 12 h (*C*_12_) being higher when the drug was given in the evening (27). This phenomenon could be due to a delayed timing of the night dose, a delayed absorption due to coadministration with food, or diurnal variation. The coadministration of abacavir with food has no effect on abacavir overall exposure (AUC) but delays abacavir absorption (29). Diurnal variation is a possible reason for the nelfinavir, ritonavir, and nevirapine morning *C*_0_ being higher than the evening *C*_10_ (30–33). The effects of the diurnal variation could be due to a decreased blood pressure and a slower pulse rate during sleep, which reduces hepatic blood flow, thereby slowing the rate of drug absorption and reducing drug metabolism (34). Since only a single predose sample was available in our study, the data were not able to reliably discriminate between the possible different options outlined above to explain the larger predose values, so we decided to use delayed absorption, which both fit the data very well and was a plausible explanation.

Our analysis has several weaknesses. Although our model can predict drug levels in the lower weight bands based on the estimated maturation effect, we had no children younger than 3 months in this study. Therefore, the drug concentrations in infants require confirmation in further studies. Additionally, the antiviral effect of abacavir is due to its intracellular anabolite, carbovir triphosphate, not measured in this study. A linear association was previously described in adults, with the intracellular levels having a longer half-life. While it is unknown whether this relationship holds in children, it suggests that using abacavir levels as a proxy is an acceptable and conservative approach (35).

To conclude, the proposed model successfully characterized the PK of abacavir, including the effect of body weight and age. Abacavir exposure was decreased by the concomitant administration of rifampin and LPV/r-4:4, but the resulting exposures were still in line with adult values, thus seemingly not requiring a dose adjustment. Higher trough concentrations were observed in the mornings, suggesting slower absorption at night (possibly due to coadministration with food) or diurnal variation. PK data in neonates and infants younger than 3 months are needed to confirm with more certainty the dose in these individuals.

## MATERIALS AND METHODS

### Study design and participants.

We conducted a prospective, open-label, one-group one-sequence study at 5 sites in 3 South African provinces: the Family Centre for Research with Ubuntu (FAM-CRU) in the Western Cape; the Empilweni Services and Research Unit (ESRU), Wits RHI Shandukani Research Unit, and the Perinatal HIV Research Units (PHRU) in Gauteng; and the Enhancing Care Foundation in KwaZulu-Natal. Enrollment occurred between January 2013 and November 2015, with the study follow-up completed in July 2016. This study is registered in ClinicalTrials.gov under identifier NCT02348177.

HIV-positive children with clinician-diagnosed TB receiving rifampin-based TB treatment could enroll if their body weight was between 3 and 15 kg and their postconceptional age was beyond 42 weeks. Children could enter regardless of whether TB treatment or ART was initiated first. We excluded children receiving nonstandard dosages of TB treatment, those requiring drugs that significantly induce cytochrome P450 enzymes, or children with clinical conditions that could compromise their study participation, such as a Division of AIDS (DAIDS) grade 3 alanine aminotransferase (ALT) level increase, renal function abnormalities, severe comorbidities, or contraindications to lopinavir-ritonavir.

All drugs, including abacavir (20-mg/ml solution), lopinavir-ritonavir-4:1 (80/20-mg/ml solution; Kaletra), and ritonavir (80 mg/ml; Norvir), were supplied through the South African Department of Health ART program. Drugs were dosed according to the study protocol (Table 4). The original protocol and amendments were reviewed by the Data Safety and Monitoring Board (DSMB) and approved by the human research ethics committees of the Universities of Stellenbosch, Cape Town, South Africa, and the Witwatersrand and Pharma Ethics in Durban, South Africa. The parents or legal guardians provided written informed consent. Consent forms were available in English and local languages, including Afrikaans, isiXhosa, and isiZulu. Due to the young age of the participants, assent was not sought.

**TABLE 4:** Antiretroviral drug dosing for children weighing ≥3 kg to <16 kg

Wt (kg)	Abacavir (mg) dose twice daily	Lopinavir-ritonavir dose (mg) twice daily	Ritonavir^*a*^ dose (mg) twice daily	Rifampin dose (mg) once a day
3–3.9	40	80/20	64	45
4–4.9	40	80/20	64	60
5–5.9	60	120/30	96	60
6–6.9	60	120/30	96	90
7–7.9	80	120/30	96	90
8–8.9	80	120/30	96	120
9–9.9	80	120/30	96	120
10–10.9	120	160/40	120	120
11–11.9	120	160/40	120	120
12–12.9	120	160/40	120	180
13–13.9	120	160/40	120	180
14–14.9	160	200/50	160	180
15–15.9	160	200/50	160	210

a Additional ritonavir used for superboosting during rifampin cotreatment and until 2 weeks after rifampin was stopped.

### Procedures.

As previously described (22), the children in the study underwent intensive pharmacokinetic sampling on three visits. The first two pharmacokinetic evaluations (PK1 and PK2) were performed, respectively, during the second and last month of TB and HIV cotreatment, which included LPV/r-4:4. Standard lopinavir-ritonavir-4:1 doses were reinstated 2 weeks after stopping rifampin, and the last sampling visit (PK3) was performed 4 to 6 weeks thereafter. On the day prior to a PK visit, caregivers were reminded to record the evening dose time and to ensure that the child fasted for at least 1 h prior to arrival at the clinic. Children were administered ART and TB drugs at the clinic and remained fasting for a further hour. Pharmacokinetic samples were drawn before the observed dose and after 1, 2, 4, 6, and 10 h. The PK visit was postponed if the participant took an incomplete dose or vomited. Safety, adherence, and virological outcomes were also assessed and have been previously reported (22).

Plasma abacavir concentrations were determined with a validated liquid chromatography (LC)-tandem mass spectrometry (MS/MS) assay developed in the Division of Clinical Pharmacology, University of Cape Town. Samples were processed with a protein precipitation extraction method using abacavir-*d*_4_ as the internal standard, followed by high-performance liquid chromatography with MS/MS detection using an AB Sciex API 3200 instrument. The calibration curves fit quadratic (weighted by 1/concentration) regressions over the range of 0.0243 to 6.21 μg/ml for abacavir. The accuracies for the abacavir assay were 104.5%, 100.6%, and 101.6% at the low, medium, and high quality control levels, respectively, during interbatch validation. The lower limit of quantification (LLOQ) was 0.0243 μg/ml.

### Statistical analysis.

**(i) Population pharmacokinetic model development.** The concentration-time data were analyzed using nonlinear mixed-effects modeling implemented in the software NONMEM (version 7.4.3) (36) with ancillary software (PsN, Pirana, and Xpose) (37). The first-order conditional estimation method with eta-epsilon interaction (FOCE-I) was used to estimate the pharmacokinetic parameters. Model building was guided by ΔOFV, proportional to −2 log likelihood; inspection of goodness-of-fit plots; VPC; biological plausibility; and clinical relevance. A decrease in ΔOFV of more than 3.84 between two nested models after the addition of one parameter was considered significant. Several structural models were evaluated, including one- and two-compartment disposition and first-order absorption and elimination with or without an absorption lag time or transit compartments. The between-subject variability (BSV), between-occasion variability (BOV), and between-visit variability (BVV) of random effects were tested on the pharmacokinetic parameters and were assumed to be lognormally distributed. BVV and BSV were tested for disposition parameters (clearance and volume parameters), while BOV was tested for absorption parameters. Each dose was treated as a separate occasion, while consecutive evening and morning doses were grouped within the same visit. To account for the uncertainty regarding the accuracy of the reported timing of the dosing on the evening preceding the pharmacokinetic visit, we tested the estimation of an additional delay in absorption for the evening dose. A combined proportional and additive error model was used to describe the residual unexplained variability. The additive error for all samples was bound to be at least 20% of the lower limit of quantification (LLOQ).

Concentrations below the limit of quantitation (BLQ) were handled by the M6 method, as described by Beal (38). Briefly, the first BLQ value after the peak (or the last in a series of BLQ values before the peak) was imputed to half the lower limit of quantification (LLOQ/2) and included in the fit with its additive error inflated by LLOQ/2, while any subsequent BLQ values (or preceding BLQ values, if the values occurred before the peak) were excluded from the fit and considered only for visual predictive check diagnostics. Allometric scaling was included to account for the known effect of body size on pharmacokinetics, with exponents fixed to 3/4 for clearance parameters and 1 for volumes of distribution (39). TBW and FFM (40) were evaluated as alternative size descriptors on each of the disposition parameters. To account for maturation, a postmenstrual age-guided function was used (equation 1):

(1) $$\text{maturation}=\frac{{\text{PMAGE}}^{\text{γ}}}{{\text{(PMAGE}}_{\text{50}}^{\text{γ}}{\text{ + PMAGE}}^{\text{γ}}\text{)}}$$

where PMAGE denotes postmenstrual age, PMAGE_50_ is the value of PMAGE at which 50% of maturation is complete, and γ is a parameter changing the shape of the relationship. Since no information on the actual gestational age of the children was available, the postmenstrual age was assumed to be the postnatal age plus 9 months. Other candidate covariates were screened based on inspection of parameter-versus-covariate plots and then tested in the model for inclusion and retained based on statistical significance (using a 3.84-point drop as significant at a *P* value of 0.05 for the inclusion of a single parameter) and physiological plausibility. The precision of the final parameter estimates was evaluated by the sampling importance resampling (SIR) method (41).

**(ii) Abacavir dosing simulations.** Monte Carlo simulations using the final model were used to simulate pediatric abacavir exposures (AUC_0–12_) during the treatment with standard lopinavir-ritonavir-4:1 and LPV/r-4:4 in children weighing between 3 and 24.9 kg under South African weight band dosing. Simulations were performed using 15,000 *in silico* patients, 100 males and 100 females at each 1-month age interval from 0 to 17 years of age. The age-weight combinations were generated from a weight-for-age model that was developed based on values from children with TB and that is hence consistent with the population for whom the dosing guidelines were designed (42). All *in silico* children were assumed to have been born at term and dosed every 12 h with abacavir. Additionally, a data set with weights between 25 and 59.9 kg was used to simulate the abacavir exposures of adolescents and adults. A once-daily dose of 600 mg was used, and AUC_0–24_ was divided by 2 to get AUC_0–12_. All simulated exposures were compared to the recommended 12-h adult median AUC of 6.02 mg·h/liter, as suggested by the European Medicines Agency (https://www.ema.europa.eu/en/medicines/human/EPAR/ziagen#product-information-section). Other adult AUC values from Yuen et al. (7), Moyle et al. (17), McDowell et al. (6), and Weller et al. (43) were also used for comparison.

### Data availability.

The data underlying the results of this study are available upon request because they contain potentially sensitive information. Interested researchers may contact the Drugs for Neglected Diseases *initiative* (DND*i*), commissioner of this study, for data access requests via email at CTdata@dndi.org. Researchers may also request data by completing the form available at www.dndi.org. In this, they confirm that they will share data and results with DND*i* and will publish any results open access.
